# A Wearable Personalised Sonification and Biofeedback Device to Enhance Movement Awareness

**DOI:** 10.3390/s24154814

**Published:** 2024-07-24

**Authors:** Toh Yen Pang, Thomas Connelly, Frank Feltham, Chi-Tsun Cheng, Azizur Rahman, Jeffrey Chan, Luke McCarney, Katrina Neville

**Affiliations:** 1 Biomedical Engineering, School of Engineering, STEM College, RMIT University, Melbourne, VIC 3000, Australia; tom.connelly@rmit.edu.au; 2 Industrial Design, School of Design, College of Design and Social Context, RMIT University, Melbourne, VIC 3000, Australia; frank.feltham@rmit.edu.au; 3 Mechanical, Manufacturing and Mechatronic Engineering, School of Engineering, STEM College, RMIT University, Melbourne, VIC 3000, Australia; ben.cheng@rmit.edu.au; 4 Occupational Health and Safety/Ergonomics, Construction, School of Property, Construction and Project Management, Design and Social Context, RMIT University, Melbourne, VIC 3000, Australia; azizur.rahman@rmit.edu.au; 5 Data Science & Artificial Intelligence, School of Computing Technologies, STEM College, RMIT University, Melbourne, VIC 3000, Australia; jeffrey.chan@rmit.edu.au; 6 Rehabilitation Sciences, School of Health and Biomedical Sciences, STEM College, RMIT University, Bundoora West, VIC 3083, Australia; luke.mccarney@rmit.edu.au; 7 Electrical & Electronic Engineering, School of Engineering, STEM College, RMIT University, Melbourne, VIC 3000, Australia; katrina.neville@rmit.edu.au

**Keywords:** sonification, gait analysis, real-time biofeedback, movement control

## Abstract

Movement sonification has emerged as a promising approach for rehabilitation and motion control. Despite significant advancements in sensor technologies, challenges remain in developing cost-effective, user-friendly, and reliable systems for gait detection and sonification. This study introduces a novel wearable personalised sonification and biofeedback device to enhance movement awareness for individuals with irregular gait and posture. Through the integration of inertial measurement units (IMUs), MATLAB, and sophisticated audio feedback mechanisms, the device offers real-time, intuitive cues to facilitate gait correction and improve functional mobility. Utilising a single wearable sensor attached to the L4 vertebrae, the system captures kinematic parameters to generate auditory feedback through discrete and continuous tones corresponding to heel strike events and sagittal plane rotations. A preliminary test that involved 20 participants under various audio feedback conditions was conducted to assess the system’s accuracy, reliability, and user synchronisation. The results indicate a promising improvement in movement awareness facilitated by auditory cues. This suggests a potential for enhancing gait and balance, particularly beneficial for individuals with compromised gait or those undergoing a rehabilitation process. This paper details the development process, experimental setup, and initial findings, discussing the integration challenges and future research directions. It also presents a novel approach to providing real-time feedback to participants about their balance, potentially enabling them to make immediate adjustments to their posture and movement. Future research should evaluate this method in varied real-world settings and populations, including the elderly and individuals with Parkinson’s disease.

## 1. Introduction

### 1.1. Sonification in Gait and Posture

Movement sonification, the process of translating movement data into auditory feedback, has seen significant advancements with the development of various sensor technologies. These advancements have facilitated many researchers to explore using music and sonification as auditory feedback to improve motor control and functional mobility in individuals with various conditions, including Parkinson’s disease (PD), gait disorders, and the consequences of neurological impairments due to illness. For example, many studies [1,2,3,4,5] have reported the capacity to use music as an external auditory cue that can help PD and post-stroke patients improve their movement control. Kantan, et al. [3] used inexpensive wireless and wearable inertial sensors to capture kinematic parameters. These parameters were then fed into software built with open-source tools to create music as sonic representation or audible sounds. Feedback to users provided via musical sonification through simple audio manipulations (e.g., pitch, volume, tone, brightness, and rhythm) has demonstrated promising results in enhancing motor learning and rehabilitation outcomes in patients with PD and stroke [1,2,3].

Raglio, et al. [4] used two inertial measurement units (IMUs) attached to each ankle of the participants to monitor heel–ground contacts during exercises. A custom MATLAB software was developed to record the heel–ground contacts and to analyse IMU-derived angles, display real-time cadence, and activate pre-recorded musical stimuli through headphones. The music-based training, which involved sonification linked to movement, was found to improve balance in patients with PD. The authors suggested that musical stimuli enhanced the predictability and regularity of movement, assisting patients to control and organise these movements.

Moreover, previous studies [1,2,4,6] have customised various musical elements into real-time auditory feedback. These approaches introduced complexity that was unhelpful for users or practitioners unfamiliar with the technologies used to create such feedback for rehabilitation outcomes. Musical sonification consists of modifying the playback of preselected music in real-time according to desired movement parameters [1,2,7,8,9]. The approach assumes a one-size-fits-all musical preference and cognitive processing capabilities. However, individual differences in musical taste and the mental load involved in processing complex musical arrangements could affect engagement and the efficacy of the intervention [10,11,12,13]. Simplifying the composition of the cues without compromising on customisation capabilities could be an area of improvement.

Besides individual musical preferences, the complexity of musical sonification poses challenges. For instance, users may need to understand the hidden relation between their actions and the resulting musical output, which could be more cognitively demanding. This complexity can be linked to understanding musical concepts such as rhythm, tonality, or pitch height, where a higher position on the musical scale corresponds to a higher or brighter pitch and vice versa [13,14]. Similarly, users must understand how their movements translate into different musical outputs, especially in the form of sonification [7,11,15].

The integration of non-musical auditory feedback in motor learning has been gaining the attention of an increasing number of sports scientists, neurorehabilitation researchers, research psychologists, and engineers due to its potential to enhance motor perception, control, and learning [4,7,16]. The use of non-musical sounds in movement training and rehabilitation requires further exploration and application of non-musical, natural sounds to enhance motor behaviour [7,9,17]. Natural sound involves focusing on those auditory cues inherently produced by the act of movement or walking. These can include the sound of footsteps, the rhythm, and the intensity of the sound, which can provide feedback on pace and heel–ground surface interaction. Such sounds offer real-time, intuitive feedback to help individuals adjust their gait for improved balance and coordination. Furthermore, Reh, et al. [14] and other researchers [18,19] have suggested that individuals can more effectively identify and associate with sound patterns produced by their movements than by others’ actions or artificial sound conditions.

The study by Linnhoff, et al. [20] explored the use of non-musical sound. Specifically, they used sinusoidal continuous tones to convey information on knee movement during gait, focusing on different phases of the walking cycle. Their approach involved mapping the knee angle to both the frequency and gain or volume of a continuous sine tone, creating auditory feedback that reflects the movement dynamics of the knee. Participants who participated in the gait analysis reported that the lower-pitched, sinusoidal continuous tones were pleasant and informative in guiding them for gait correction and training.

Recently, Feltham, et al. [21] developed a sonification system that combined continuous and discrete auditory tones to reflect changes in the person’s movements, specifically focusing on rotational deviations within the sagittal plane. A MATLAB (R2022b) code was developed to detect movement and integrate with a Max (version 8) software for sonification. The continuous auditory feedback consisted of tones that varied in ‘brightness’ or pitch based on the person’s rotation relative to a pre-defined reference region. As the person moved further from the reference point, tones brightened, signalling increased displacement from this reference point. Conversely, the tones dulled with movement around the reference region, providing an intuitive cue indicating reduced displacement. They also incorporated a discrete bell tone, which served as an alert mechanism when the individual moved outside the reference region. The continuous and discrete tones provided clear, straightforward feedback without the complexity of musical structures and motifs that may be distracting. Thus, future research and development efforts should focus on creating adaptable, user-friendly systems that can be customised to individual needs, thereby maximising therapeutic outcomes in gait and posture rehabilitation.

### 1.2. Sensor Technologies and Techniques for Motion Detection and Sonification

Numerous wearable devices are capable of detecting and tracking the dynamic motion in human limbs [11,22,23]. However, we have narrowed our scope to review relevant techniques and sensors that specifically address gait event detection and provide auditory feedback through movement sonification (Table 1).

Many of the reviewed studies have used sampling rates between 100 Hz [20] and 1000 Hz [16], and those with high sampling rates faced challenges in real-time applications. Previous studies [20,25,26] have utilised advanced IMU systems, which managed to capture detailed biomechanical and kinematics data. For example, Linnhoff, et al. [20] developed a Python code to sonify the collected sine continuous waveforms, Reh, et al. [25] developed a MATLAB algorithm to detect gait events, and Teufl, et al. [26] used retroreflective markers with optical cameras to capture accurate joint kinematics. Their approaches required complex and costly setups that depended on sophisticated equipment and dedicated laboratory space.

It is important to note that this is not an exhaustive list, and many other methods and technologies not covered here may also contribute valuable insights and advancements in this field. However, these studies reveal challenges for the practical application of movement sonification technologies, including the need for simplified, cost-effective solutions suitable for diverse environmental conditions and user groups.

### 1.3. Real-Time Biofeedback and Personalised Cueing

As described in the study by Raglio, et al. [4], sonification can be customised to suit each patient’s individual needs and preferences. This includes adjusting the sound parameters (e.g., pitch, volume, or rhythm), mapping, and feedback to best align with the rehabilitation goals and the patient’s capabilities. The customisation was also agreed upon by Reh et al. [7] and Reh, et al. [14], who stated that a deep connection exists between an individual’s motor actions and the auditory feedback those actions can generate. Wall, et al. [15] also suggested personalising sonification to an individual’s physiological condition rather than a one-size-fits-all approach. For example, Raglio, et al. [4] used pre-recorded musical stimuli that were subsequently produced in real-time in relation to the heel–ground contact position of a patient. Tailoring the musical pattern to align more closely with individual patient preferences increases the perceived value and effectiveness of sonification-based rehabilitation. However, ensuring instantaneous and accurate feedback remains challenging, requiring sophisticated software to interpret physical movements into meaningful auditory cues.

IMU-based real-time biofeedback systems, such as the “sofigait” prototype by Linnhoff, et al. [20], synchronise acoustic feedback with the user’s knee-angle motion, thus providing immediate cues for gait correction and enhancement. Despite promising results, their system showed discrepancies in measurement accuracy compared to gold-standard optoelectronic camera-based systems. Hence, moving forward requires researchers to improve their sensor technology and algorithm development to minimise errors and optimise the real-time processing of kinematic data.

Building on the foundational work of Rodger et al. [16,27] in balance and posture, Feltham, et al. [21] developed an auditory feedback system through an IMU sensor that was attached to the participant. The sensor was used to capture real-time movements. It triggered either a discrete bell tone to indicate significant deviations from the balance threshold or modulated the pitch of a continuous tone for minor adjustments. By setting thresholds that reflect individual balance capabilities and providing feedback tailored to these parameters, the study leveraged the ability of participants to respond more effectively to sounds generated by their movements. Integrating personalised auditory cueing and real-time biofeedback has offered an intuitive means for participants to recognise and correct their balance in real-time.

Despite the promising outcomes of using complex and continuous audio (bio-) feedback to enhance the sense of control and awareness over one’s movements [8,28], several challenges exist, including the need for more robust and reliable biofeedback solutions. In order to address the limitations of previous technologies, e.g., system synchronisation and offer personalised feedback cues based on their movements, this study aimed to develop a more accurate and reliable algorithm for gait event detection using single wearable sensing technology.

## 2. Materials and Methods

### 2.1. Design Considerations and Development

**Figure 1:**
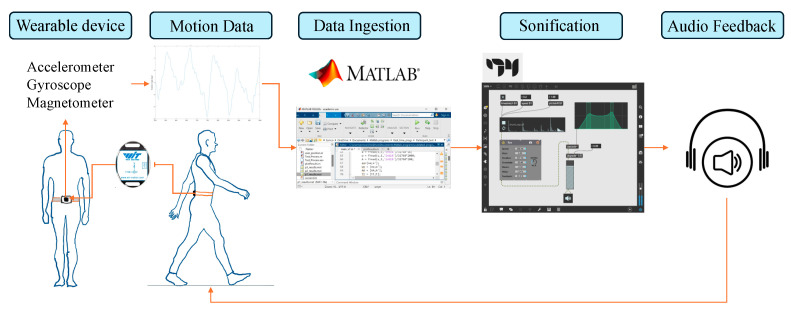
A wearable system features a single IMU sensor secured in a small pocket of a belt-like garment positioned at the user’s back. MATLAB was used to process the incoming data from the IMU and transmitted the extracted samples for queries on the read path to Max MSP, which then produced auditory cues in the form of real-time feedback to users. For a more personalised experience, users may opt for either a headset or a speaker depending on their preferences and circumstance.

The system was developed using a single IMU sensor held in place by a wearable garment (see Figure 1). The garment was attached at the waist like a belt, with the sensor placed in a small pocket, positioning the sensor at the user’s back (L4 vertebrae). The IMU sensor adopted in this study was the WT901BLE from WitMotion (Shenzhen Co., Ltd., Shenzhen, China). It is a lightweight (50 g) IMU sensor equipped with a 3-axis XYZ configuration for measuring Pitch, Roll, and Yaw tile angles. The sensor was configured to transmit data at 100 Hz. It also includes features for acceleration, gyroscopic motion, and magnetic field assessment.

### 2.2. Software and Processing Unit

**Figure 2:**
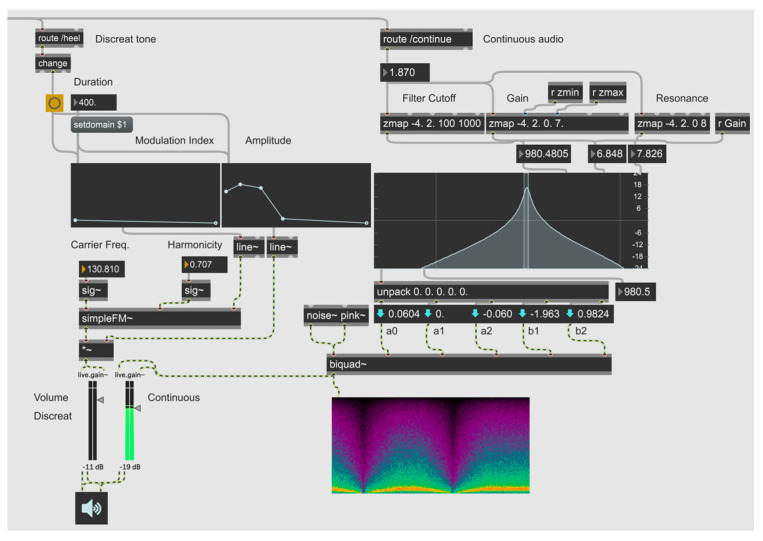
Schematic representation of the modulation of Open Sound Control (OSC) protocols in the local TCP/IP connection between MATLAB and Max MSP, leading to the generation of a discrete tone upon heel strike detection.

**Figure 3:**
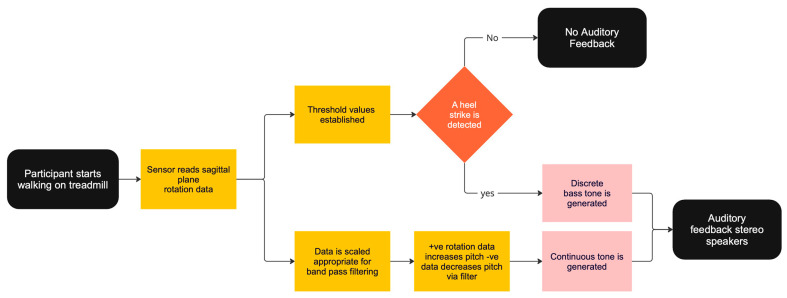
State diagram of the logic driving the sonification used for auditory feedback.

The sensor was connected to a laptop via a USB-C cable to yield a stable and high data transmission rate. MATLAB (R2022b) was used as a data ingestor to read and record the sensor’s angular data. MATLAB also processed real-time data to detect participants’ heel strikes. The features extracted from the raw data were then transmitted to Max MSP, an audio processing software, to synthesise sonification signals by ^©^Cycling’74. MATLAB and Max MSP (version 8) were connected locally via TCP/IP and the open sound control communication (OSC) protocols (Figure 2). MATLAB conveyed processed sensor data to Max MSP during program execution, to generate the corresponding discrete tone when a heel strike was detected. Depending on the test condition, MATLAB also sent angle rotation information of the sagittal plane to Max MSP, to generate the continuous audio signal (Figure 3).

### 2.3. Personalised Cueing and Audio Feedback

Comprehensive information about the design and configuration of the auditory feedback system, including the interactions among the IMU sensor, MATLAB, and Max MSP, has been published previously [21,29].

**Figure 4:**
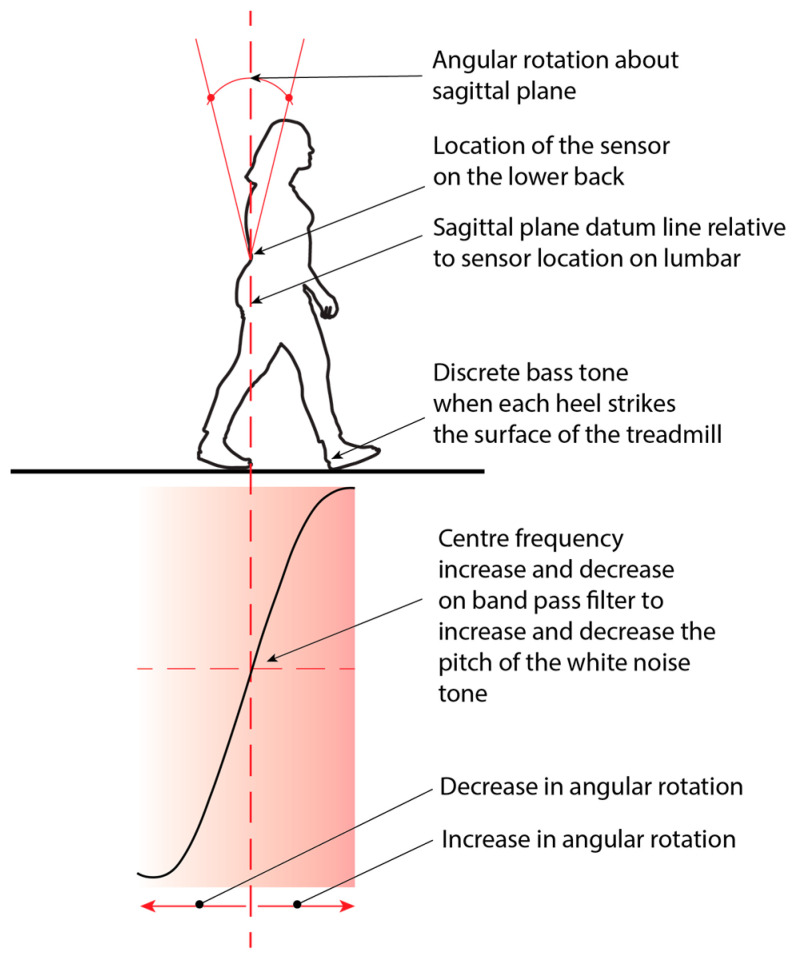
The relationship between rotation movements on the sagittal plane, with the sensor located on the L4 vertebrae, and its subsequent increase or decrease in the centre frequency of the noise generation. The rotation increase raised the pitch, and the decrease lowered the pitch of the white noise or wind-like auditory feedback.

The IMU sensor that was positioned on the lower back (e.g., L4 vertebrae) was used to measure rotation movements on the sagittal plane. MATLAB was employed to calculate threshold values specific to each user, used for detecting heel strikes. The detection process involves analysing the relationship between these rotational movements and the subsequent increase or decrease in the centre frequency of noise generation (Figure 4). For this study, discrete and continuous tones were generated within the Max MSP software environment. The discrete tone, synthesised as a frequency modulation (FM) bass tone, was triggered when MATLAB identified a heel strike, subsequently initiating the audio output.

The continuous tone was generated by Max MSP. This was achieved when MATLAB sent the rotation angle on the sagittal plane to proportionally adjust a bandpass filter’s frequency, amplitude, and resonance over a white noise generator. This method of filtering white noise created a wind-like sound based on the swing phase of the participants’ gait cycle. The bass and wind-like sound were amplified through either speakers or headphones to provide real-time feedback to participants. The auditory feedback approach was designed with flexibility in accommodating different user preferences and circumstances.

### 2.4. Sample Size

The sample size was determined based on theoretical guidelines and practical constraints. Guest, et al. [30] suggested data saturation occurs after 12–15 participants, while Crouch, et al. [31] recommended 20 homogeneous participants for sufficient data saturation and accounting for outliers. Given these guidelines, along with our limited timeframe, limited funding available (which restricted our ability to support a larger participant pool), and the workload and availability of the investigators involved in managing this study, we determined that a sample size of 20 participants was appropriate and sufficient for the objectives of this study.

### 2.5. Experimental Setup and Data Processing

**Figure 5:**
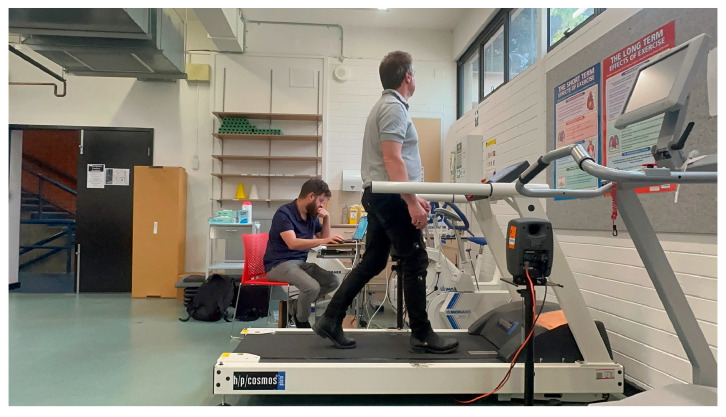
The experimental setup in RMIT University’s Biomechanics lab shows a participant performing walking with the wearable system positioned on their lower back and auditory feedback provided through speakers on either side.

The experiment in this study involved 20 participants and was conducted in the Biomechanics Laboratory of RMIT University. This study was conducted according to the guidelines in the Australian National Statement on ethical conduct in human research and the Australian Code for the Responsible Conduct of Research. It had ethical approval from the College Human Ethics Advisory Network committee. All participants signed an informed consent form prior to participating in the experiments. The experiment involved participants performing a gait analysis with the wearable system positioned at their lower back and walking comfortably on a treadmill (Figure 5).

Recognising the importance of gait adaptation to walking pace, we ensured that each participant spent a minute or two walking on the treadmill with a comfortable pace, to ensure that participants reached a natural and stable gait. During this time, participants could adjust to their movement and gait in order to establish a consistent walking rhythm.

Once participants had familiarised themselves with the treadmill environment, the actual data collection began. Participants were asked walked at two different speeds, i.e., 2 km/h and 3.9 km/h, with three audio configurations. Sonification feedback was provided through speakers located on either side of the treadmill. In the first configuration, participants walked without any audio being generated. This test was conducted to establish and record a baseline. During the no-audio test conducted, threshold values were calculated in the MATLAB program; it was found that this value was unique for each participant.

The second configuration gave participants discrete audio tones generated when a heel strike was detected. The detection process involved sampling the rotation angles on the sagittal plane to identify turning points in the time series data such that

$$x=\left(a\left[n-1\right]-a[n-2]\right)\times \left(a\left[n-2\right]-a[n-3]\right),$$

where *a*[*n*] represents the *n*-th sample of the rotation angle on the sagittal plane, and *x* is an indicator that will return a negative value when a turning point is located at *a*[*n* − 2] and vice versa. The following expression was further used to determine the trend across the *n* − 1-th and *n* − 2-th samples, such that

$$y=\left(a\left[n-1\right]-a[n-2]\right).$$

Here, *y* returns negative values for a decreasing trend and vice versa. Combining *x* and *y* allows one to identify a positive local peak from a negative one in time-series data without performing differentiations. A positive value of *xy* indicated a positive peak at *a*[*n* − 2] and vice versa. A threshold was established to reject local peaks with magnitudes too close to their adjacent peaks, which ensures that only significant peaks are considered, enhancing the accuracy of heel strike detection. The threshold was derived from the no-audio test.

The third configuration provided participants with continuous audio feedback. Processed sensor data were streamed to Max MSP to vary the bandpass filter parameters. As mentioned above, the combination of the signal generator and the bandpass filter created a continuous wind-like sound based on participants’ movements.

Each audio condition was tested three times for 15 s each at both speeds, resulting in 18 data sets for each participant.

### 2.6. Data Analysis

After the walking experiment, participants were asked to provide subjective feedback on their experience during the movement activities and how the sonification feedback influenced their movement. The *t*-test or the Wilcoxon signed-rank test was employed for statistical analysis, based on whether the data followed a normal distribution. A *p*-value of less than 0.05 was deemed statistically significant within a 95% confidence interval. A thematic analysis was performed on the qualitative data, where the interview transcripts were examined to determine meaningful themes and patterns.

## 3. Results

The results presented in this section were collected from 20 participants (12 male and 8 female, mean age = 35.5 ± 10.2) in the experiment. All participants self-reported as healthy and did not undergo any major lower limb surgeries in the three months prior to taking part in the experiment.

### 3.1. Evaluation of the System

**Figure 6:**
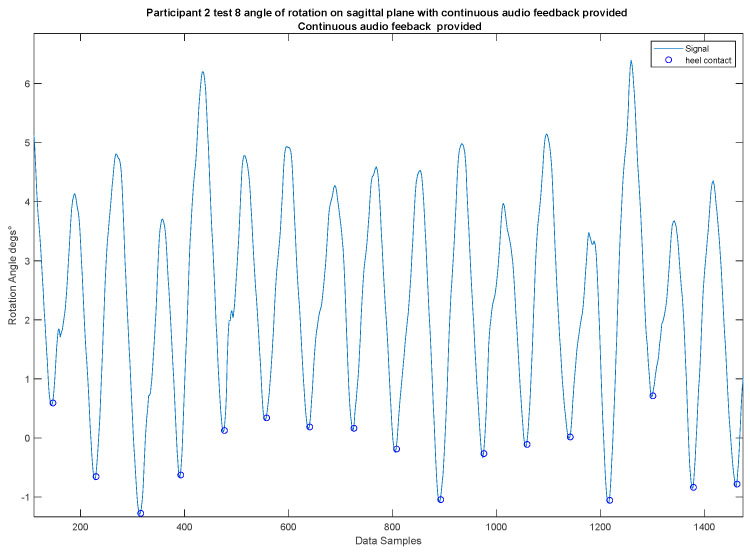
Visual representation of a heel strike event. The figure highlights the symmetrical swing of the CoG oscillating between legs contacting the ground (blue circular symbols), which triggers the generation of audio cues.

The system provided audio cues to guide participants to have a more symmetrical swing while the centre of gravity (CoG) oscillated between the legs. When a participant leaned to one side for any reason, they would not receive audio cues for both heel strikes. However, when a participant demonstrated a more symmetrical swing of the CoG, oscillating evenly between their legs and contacting the ground, they would receive audio cues for both heel strikes (Figure 6).

We evaluated the effectiveness of the proposed auditory feedback system in assisting participants in achieving a more symmetrical swing of their CoG while walking. The system provided audio cues at the maximum and minimum points of the swing waveform, aligning closely with the participant’s heel strikes. If participants leaned to one side due to dominant leg usage or health issues, they may have missed some audio cues, prompting them to adjust their posture for balanced walking.

**Figure 7:**
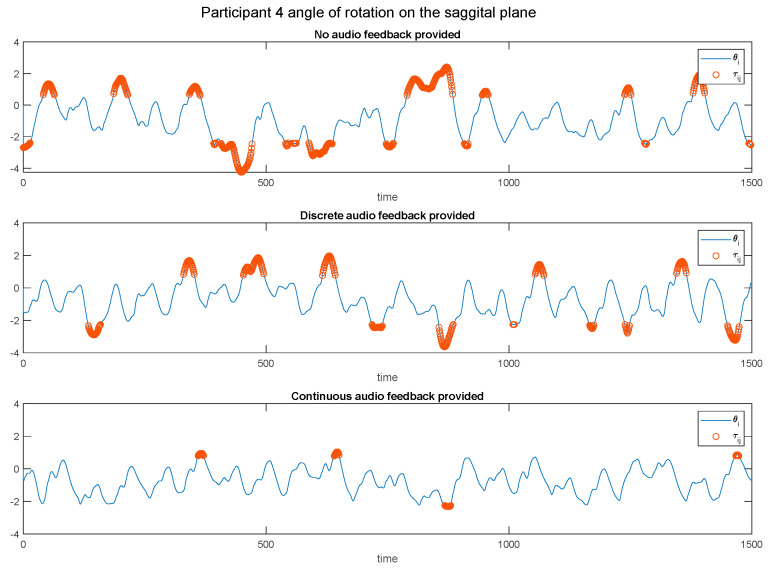
Graphical representation of a participant’s balance improvement over time. The graph initially shows significant off-balance movements, as indicated by the waveform where the participant leans to one side. Towards the end, the graph demonstrates the participant’s improved adjustment of their CoG, with less swing to either side, indicating the effectiveness of regular audio cues.

The improvement in participants’ walking posture was assessed by analysing the changes in the leg swing waveform over time (Figure 7) (for a detailed representation of individual participant data, please refer to Table A1 in Appendix A). At the beginning of this study, participants often received fewer audio cues due to an uneven CoG, showing a bias towards the left or right leg. As participants adjusted their posture in response to the feedback, the frequency of received cues increased, indicating a more balanced CoG.

**Figure 8:**
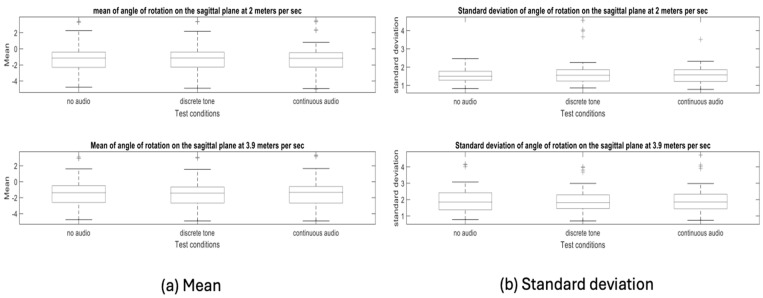
Comparative graph illustrating (**a**) the mean and (**b**) standard deviation of the 20 participants’ walk at the baseline (silent walk) and when auditory cues were provided.

Initially, the waveform showed a significant bias, correlating with the missed audio cues. By the end of this study, the waveform average shifted towards the centre, demonstrating that participants had achieved a more balanced CoG and were consistently receiving cues for both heel strikes. To quantify this improvement, we compared the mean of the swing waveform for the baseline (silent walk) with the auditory cues (Figure 8). For the mean angle of rotation on the sagittal plane of individual participant data, please refer to Table A2 in Appendix A.

This change highlights the system’s effectiveness in enhancing participants’ awareness of their walking posture through sonification. Participants could adjust their CoG without explicit instructions about the audio cues’ meaning, showcasing the feedback mechanism’s intuitive nature.

### 3.2. User Feedback on the Awareness of Movement Due to Sonification

**Figure 9:**
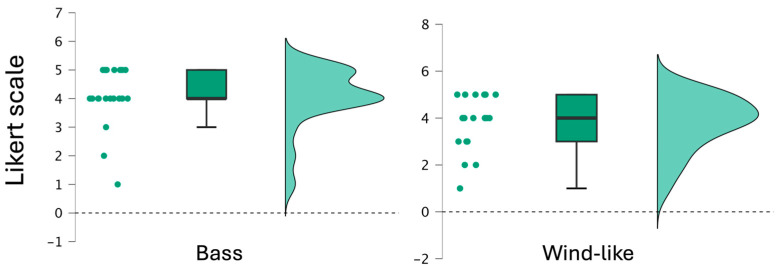
The distributions of responses of the two Likert-scale (on a scale from 1 (very unaware) to 5 (very aware)) survey questions about bass and wind-like audio cues.

The research presented in this section aimed to determine whether the proposed sonification feedback system made participants more aware of their movements and to identify where in the body this awareness occurred. The mean, standard deviation, and distribution for each Likert-scale question related to the bass and wind-like audio cues are shown in Figure 9. This visual comparison highlights the overall similarity in response patterns between the two questions.

The responses to the Likert-scale questions did not follow a normal distribution, so a Wilcoxon signed-rank test was used to compare the responses and assess whether there were significant differences in the participants’ answers. The Wilcoxon signed-rank test showed no statistically significant difference between the bass and wind-like cues (Z = −3.36, *p* = 0.37).

Some common themes from the participants’ responses are the following:

- Awareness of movements: Many participants (70%) reported increased awareness of their movements, particularly at the beginning of their footstep cycle, which is often linked to the heel strike and midfoot stance phases of their gait. About 50% of the participants felt that the sonifications influenced their footstep timing and gait, making them more conscious of their heel strikes and overall walking patterns.
- Initial confusion and adaptation: Some participants (60%) initially found the sound confusing due to its lack of a fixed rhythm, which affected their engagement with the system. However, 40% of the participants found themselves subconsciously adjusting their steps to align with the audio cues.
- Personal sound preferences: Several participants (45%) indicated that personal sound preferences might have influenced their experiences when processing the audio cues and affected their engagement.

## 4. Discussion

The wearable sensor presented in this study, which uses IMU features with accelerometers and gyroscopes, is seamlessly attached to the body and provides continuous gait monitoring. This sensor is non-intrusive and can offer real-time feedback for users, who synchronise their movements to a rhythmic auditory cue, as recommended [3,5]. The biofeedback signal in our study is a sonified signal, where white noise is distorted based on the readings from the sensor. The signal is then amplified through either speakers or headphones to provide real-time feedback to participants. This auditory feedback helps participants adjust their movements based on the detected rotational movements on the sagittal plane, as measured by the sensor on their lower back. Our proposed method addressed the challenges faced with expensive and sophisticated equipment (Table 1) by using a single low-cost sensor to achieve reasonable gait event detection, aiming to balance affordability, ease of use, and data collection and sonification reliability. Our approach aimed to provide a more accessible solution for gait analysis and movement sonification, paving the way for broader applications in clinical and everyday usage settings.

### 4.1. System Integration

Integrating sensor data with an audio feedback system in gait analysis and rehabilitation presents several technical and user-experience challenges that must be addressed to ensure effectiveness and reliability. Initially, we planned to use Bluetooth on the IMU to transmit data wirelessly. However, we encountered connectivity issues, with the signal frequently interrupted. This inconsistency in data transmission presented a risk of losing kinematic data, which could negatively impact the effectiveness of the audio feedback. To resolve these dropping-off issues, we switched to a cable connection, which provided a stable and reliable data transmission pathway, ensuring no data were lost during the experiments.

### 4.2. Algorithmic Approach for Sonification

Developing algorithms for heel strike detection and sagittal plane rotation is a critical component of our audio feedback system for gait analysis. These algorithms have been evaluated based on detection accuracy, responsiveness, computational efficiency, and adaptiveness to rapid movement changes. The algorithms were designed to process real-time data inputs and provide immediate auditory feedback. They demonstrated high accuracy in detecting heel strikes and capturing sagittal plane rotation during the pilot test and subsequent experiments with 20 participants. This real-time capability and the accurate identification of heel strikes, in particular, ensure that the feedback is synchronised with the user’s step cycle and can enhance the user’s awareness of their gait.

### 4.3. Evaluation of the System and User Engagement

Two synthesis strategies were developed to provide participants with immediate auditory feedback based on real-time positing of the heel striking the ground. It was reported that the type of sound and its presentation significantly influence its effectiveness. The initial confusion reported by participants in the current study emphasises the need for carefully designed auditory cues that match the natural rhythm of walking.

Reflecting on the participants’ feedback, it appears that the awareness and usefulness of the auditory feedback varied among individuals. Some participants found the auditory cues helpful in increasing their awareness of their movements, particularly at the beginning of their footstep cycle. However, others found the sound confusing or distracting, particularly when it did not align with their pace or rhythm of walking. Such observations indicated that personal expectations and preferences can influence the user’s engagement with the system and its perceived effectiveness.

Our findings contribute to the understanding that sonified signals, in the form of non-musical sounds, can influence movement in a healthy population. The analysis showed that participants who received sonified feedback exhibited better gait stability and coordination, i.e., achieving a more symmetrical swing of their CoG than those without any auditory cue. This aligns with Effenberg, et al. [7], who demonstrated that movement sonification supports motor control by providing additional real-time auditory feedback that integrates with proprioceptive feedback.

Overall, the feedback highlights both the potential and the limitations of using sonification for movement awareness. While the irregular rhythm posed an initial challenge, the sound ultimately increased participants’ awareness of their movements. These findings suggested that with adjustments to the rhythmic consistency of the auditory feedback, sonification can offer a valuable tool for enhancing movement awareness and improving gait patterns, which concurs with previous studies [14,17,19,20].

### 4.4. Limitations and Future Works

In the experiments, the algorithms occasionally failed to capture the kinematic events accurately, especially with sudden accelerations or decelerations. This limitation was particularly relevant when participants suddenly changed their walking speeds. The inability to consistently track rapid movements can lead to delays or inaccuracies in the auditory feedback generation, potentially confusing the users.

While the auditory feedback system showed potential in increasing movement awareness for some participants, the personal sound preferences associated with processing audio cues presented challenges that need to be addressed to enhance the system’s intuitiveness and usefulness.

Our study presents a step forward in the development of an accurate and reliable algorithm for gait event detection using single wearable sensing technology. However, we acknowledge that the present solution may pose challenges when adapting to populations with neurological conditions. These individuals may have auditory problems or difficulties integrating sensory–motor responses in addition to cognitive disorders. As such, future research should consider these potential challenges.

A future direction for this research is to explore the application of this method in diverse and complex real-world environments, including outdoor settings and varied terrains, to further validate its robustness and application in everyday use. Additionally, expanding the study to include a wider population, such as elderly individuals and people living with PD, will allow for investigating its potential for enhancing motor function control and rehabilitation in broader contexts.

In addition to the audio feedback, integrating a vibratory stimulus, such as haptic feedback, could potentially aid those with auditory difficulties and enhance the overall effectiveness of the device for gait rehabilitation. This opportunity warrants exploration in future studies.

Despite efforts to optimise the algorithms, the presence of signal noise from the IMU sensors occasionally impacted the accuracy of the detections. The sensor placement and individual variations in walking style were also found to introduce noise into the data, indicating that there is still room for improvement to enhance the algorithm’s robustness. Further research and development should focus on refining the algorithms to better accommodate variability in gait patterns and improve robustness against noise and rapid movement changes.

Future work should also aim to develop a smaller, more portable device with lower processing requirements, such as utilising a low-cost single-board computer, like Raspberry Pi, or packaging it into a mobile phone application. This approach could enhance the practicality and accessibility of the system, making it easier for users to incorporate it into their daily routines without needing a laptop or other bulky equipment.

## 5. Conclusions

This research introduces an innovative approach to enhancing movement awareness through a wearable personalised sonification and biofeedback device. The wearable sensor, which uses IMU features, is seamlessly attached to the body, providing real-time feedback by synchronising users’ movements with a rhythmic auditory cue. The current study addresses key challenges identified in existing movement sonification and gait analysis technologies, such as high complexity, extensive setup requirements, integration challenges, and significant costs. By using a single low-cost sensor for gait event detection, our proposed method simplifies the setup. It reduces the overall cost, making the technology more accessible and practical for wider applications, including movement awareness and rehabilitation. The primary advantage of the cue is its potential to provide immediate, intuitive feedback without requiring complex equipment and with a relatively short learning phase.

However, to maximise its effectiveness, the rhythmic consistency of the auditory feedback needs improvement to align more closely with natural gait patterns. Nonetheless, the system offers a significant advancement in rehabilitating individuals with irregular gait and posture by providing real-time auditory feedback based on kinematic data. The initial findings demonstrate the device’s potential to improve functional mobility, indicating a promising future research and development avenue. To fully realise its potential, future research should focus on applying this method in diverse and complex real-world environments. This includes a wider population, such as elderly individuals and those living with PD, and indoor and outdoor settings, to assess its robustness and application for daily use.

## Figures and Tables

**Table 1:** Various sensor technologies and approaches used in movement detection and sonification in previous studies.

Technique	Sensors	Motion Detection and Sonification	Advantages and/or Disadvantages
Analog system [24]	Analog gyroscope and accelerometers	Oqus camera system	High resolution with oversampling, detailed motion analysis Wired setup, limited mobility, complex data processing
Ground reaction force [16]	AccuGait force-plate	Qualisys Oqus-3 cameras	High accuracy in ground-reaction force measurement Complex setup, high cost, requires dedicated laboratory space
IMUs Feltham, et al. [21]	A single IMU (integrated 3-axis gyroscope, 3-axis accelerometer, and 3-axis magnetometer)	MATLAB code	IMU sensor communicates wirelessly and provides real-time audio feedback Simple setup, just used to detect balance control
Linnhoff, et al. [20]	Four IMU sensors (integrated triaxial accelerometer, triaxial gyroscope, magnetometer, and microcontroller)	Python code	Real-time acoustic feedback, validated by Vicon Motion capture, detailed knee angle measurement Complex setup requiring rigid boxes for attachments, potential for data drift
Raglio, et al. [4]	Two IMU sensors (integrated accelerometers and gyroscopes)	MATLAB code	Non-restricting, lightweight, wireless devices that can be worn on the body Pre-recorded musical stimulus, false positive and false negative feedback
Reh, et al. [25]	System of seven wireless inertial sensors	MATLAB code	Acoustic real-time feedback, detailed temporal gait parameters measurements Complex setup, dependent on software and hardware integration, steep learning curve for users
Teufl, et al. [26]	Seven IMU sensors (integrated gyroscopes and accelerometers)	Optical cameras	Joint kinematics were validated by OptiTrack Prime cameras Complex setup, no sonification feedback

## Data Availability

The data presented in this study are available on request from the corresponding author. The data are not publicly available due to privacy.

## Appendix A

**Table A1:** Participants’ data demonstrating the percentage of uneven swing of their centre of gravity while walking under different auditory cue conditions.

Participant ID	No Audio	Discrete	Continuous
1	30.93%	28.80%	33.60%
2	45.00%	45.60%	48.00%
3	39.93%	40.40%	47.67%
4	27.13%	17.33%	2.87%
5	49.33%	46.20%	51.60%
6	25.07%	35.40%	29.00%
7	21.80%	17.40%	18.87%
8	22.67%	16.53%	10.33%
9	22.00%	37.33%	32.40%
10	35.40%	37.60%	30.07%
11	13.40%	11.73%	18.27%
12	8.47%	13.93%	17.40%
13	46.00%	44.80%	49.00%
14	39.13%	38.13%	47.27%
15	35.60%	34.53%	38.73%
16	62.07%	48.47%	49.33%
17	37.27%	41.27%	34.73%
18	45.33%	46.67%	41.40%
19	41.20%	47.13%	40.27%
20	32.53%	31.47%	40.80%

**Table A2:** The comparison of mean values of angle of rotation from the sagittal plane for the 20 participants walking in different auditory conditions.

Participant ID	Walking Speed					
	2 km/h			3.9 km/h		
	No Audio (°)	Discrete (°)	Continuous (°)	No Audio (°)	Discrete (°)	Continuous (°)
1	0.71572	0.79182	0.80502	1.35652	1.1808	1.17453
2	2.16181	2.09157	2.34176	1.54489	1.65214	1.51026
3	3.34663	3.38363	3.4305	2.99839	3.26405	3.02818
4	−0.86546	−0.8696	−0.89281	−0.67104	−0.73644	−0.79188
5	−0.81115	−0.67619	−0.72091	−0.3834	−0.54749	−0.62744
6	−2.51562	−2.54313	−2.41103	−2.38821	−2.50841	−2.49048
7	−1.16815	−1.12697	−1.05428	−1.21995	−1.17348	−1.11223
8	−1.5885	−1.52865	−1.43568	−1.53717	−1.61223	−1.55882
9	−0.29455	−0.1816	−0.44196	−0.83046	−0.83082	−0.76341
10	−2.13539	−2.18334	−2.17401	−2.80198	−2.88276	−2.86627
11	−0.54938	−0.59242	−0.54363	−0.87196	−1.00595	−0.91564
12	−2.39685	−2.55208	−2.61632	−3.3719	−3.34896	−3.22874
13	−1.23542	−1.33203	−1.21123	−1.58142	−1.48197	−1.60164
14	−2.01704	−1.92003	−1.82618	−2.41333	−1.9952	−2.1423
15	−1.2268	−1.14859	−1.3043	−1.4874	−1.54337	−1.61742
16	−0.99331	−0.9177	−0.85362	−1.16707	−1.19621	−1.26316
17	0.08646	0.09898	0.03862	−0.08541	−0.03387	0.07757
18	−4.63385	−4.81796	−4.87633	−4.68477	−4.83437	−4.81773
19	−3.58613	−3.514	−3.54151	−3.77229	−3.95034	−3.83729
20	−4.12038	−4.28964	−4.20403	−4.39558	−4.42439	−4.22397
